# Association between Human Genetic Variants and the Vaginal Bacteriome of Pregnant Women

**DOI:** 10.1128/mSystems.00158-21

**Published:** 2021-07-20

**Authors:** Wei Fan, Hui Kan, Hai-Yan Liu, Tian-Lei Wang, Yi-Ning He, Miao Zhang, Ya-Xin Li, Yi-Jie Li, Wei Meng, Qing Li, An-Qun Hu, Ying-Jie Zheng

**Affiliations:** a Department of Epidemiology, Fudan University, Shanghai, China; b Key Laboratory for Health Technology Assessment, National Commission of Health and Family Planning, Fudan University, Shanghai, China; c Key Laboratory of Public Health Safety, Ministry of Education, School of Public Health, Fudan University, Shanghai, China; d Department of Clinical Laboratory, Anqing Municipal Hospital, Anqing, China; e Department of Obstetrics and Gynecology, Anqing Municipal Hospital, Anqing, China; University of Georgia

**Keywords:** pregnant women, vaginal bacterial traits, genetic variants, bacteriome, microbiome, 16S amplicon

## Abstract

The influence of human genetic variants on the vaginal bacterial traits (VBTs) of pregnant women is still unknown. Using a genome-wide association approach based on the 16S rRNA bacteriome analysis, a total of 72 host genetic variant (single nucleotide polymorphisms [SNPs], indels, or copy number variations [CNVs])-VBT associations were found that reached the genome-wide significance level (*P* < 5 × 10^−8^) with an acceptable genomic inflation factor λ of <1.1. The majority of these SNPs that reached the genome-wide significance level had a relatively low minor allele frequency (MAF), and only seven of them had MAFs greater than 0.05. rs303212, located at the IFIT1 gene on chromosome 10, was the most eye-catching variant, which had a genome-wide association with the relative abundance (RAB) of *Actinobacteria* and *Bifidobacteriaceae* and also had a suggestive association with the RAB of a few common vaginal bacteria including *Actinobacteriota*, *Firmicutes*, *Lactobacillus*, and Gardnerella vaginalis and the beta diversity weighted UniFrac (*P* < 1 × 10^−5^). The findings of the study suggest that the vaginal bacteriome may be influenced by a number of genetic variants across the human genome and that interferon signaling may have an important influence on vaginal bacterial communities during pregnancy.

**IMPORTANCE** Knowledge about the influence of host genetics on the vaginal bacteriome in pregnancy is still limited. Although a number of environmental and behavioral factors may exert influences on the structure of vaginal bacterial communities, the vaginal bacteriome often undergoes a relatively fixed transition to a more stable and less diverse state as the menstrual cycle stops, which raises questions on the effects of human genetics. We utilized a genome-wide approach to identify the associations between genetic variants and multiple VBTs and performed enrichment analyses. The human genetics during pregnancy may be involved in multiple pathways. The results may disclose innate functional factors involved in shaping the vaginal bacteriome during pregnancy and provide insight into the establishment of specific strategies for prevention and clinical treatment of pregnancy complications.

## INTRODUCTION

Commensal bacteria thriving on various human body sites are believed to contribute to host nutrition, protection from pathogens, or the development of an immune response (1, 2). The same is true for the vaginal bacteriome. The bacterial ecosystem and multiple predisposing factors shaping its structure are critical to the biological pathways leading to internal homeostasis or disorders (3, 4). Disturbances in the vaginal bacteriome may contribute to a variety of common obstetric or gynecological disorders including bacterial vaginosis (BV), preterm birth, and other infectious diseases (5–8).

Pregnancy is a special phase in a woman’s life that involves many physiological changes from physical to hormonal (9). The vaginal bacteriome often undergoes a pronounced and relatively fixed change during pregnancy to a more stable and less diverse state in response to these changes as the menstrual cycle stops (8). Previous studies have suggested that the composition of the commensal vaginal bacteriome can be determined by multiple behavioral and environmental factors including sexual behavior, pregnancy, smoking, and diet (10–12). Although these studies have uncovered several lines of host-bacteriome interactions, the reasons for the relatively fixed transition of the vaginal bacteriome upon pregnancy remain unclear (13–15). In light of the complex etiology of microbial traits, questions about host genetics are raised to fill the paucity of intrinsic determinants of vaginal bacterial ecosystems.

Humans harbor bacteria that may acquire beneficial functions in response to host adaptation to local environments under the pressure of natural selection and become heritable in the host population when specific genetic variants evolve to replace or recruit these beneficial bacteria (13, 16). Empirical genome-wide association studies (GWAS) in recent years have suggested that the influence of host genetic variants on the human bacteriome can be body site specific and vary by ethnicity (14, 17–21). However, the influence of host genetic variants on the vaginal bacteriome in pregnancy has scarcely been addressed among these studies. As vaginal microbiota can aid maternal receptivity and promote a tolerogenic maternal immune system for a successful pregnancy, candidate gene-based association studies have often concentrated on the role of immune-related genes like interleukin-1 receptor antagonist gene and Toll-like receptor 4 (TLR4) gene in its colonization (9, 22–24). Evidence has confirmed the functional roles of several immunomodulators in the molecular mode of pathogen-associated molecular patterns (PAMP) in response to the vaginal bacterial communities, but genome-wide evidence of the effects of genetic variants on the structure of the vaginal bacteriome in pregnancy is still limited.

Given that the potential function of the host-vaginal bacteriome relationship could help stabilize the vaginal bacterial ecosystem and the pathogenesis of pregnancy complications, we performed a GWAS based on the 16S rRNA gene-targeted bacteriome analysis to determine whether host genetic variants are associated with multiple vaginal bacterial traits (VBTs) with the baseline data in a cohort of pregnant Chinese women.

## RESULTS

### Overview of VBTs in pregnant women.

**FIG 1:**
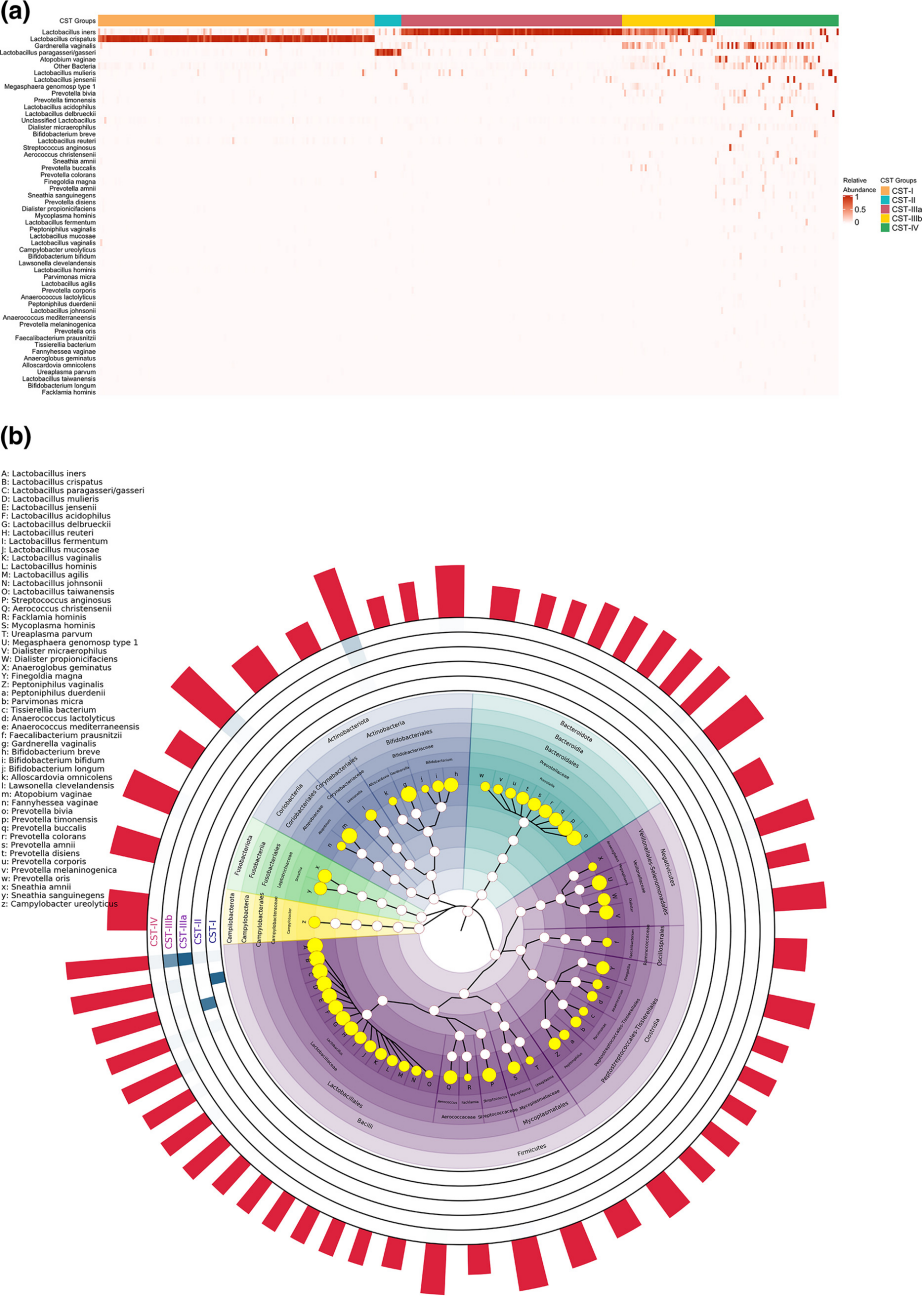
Heatmap and cladogram of vaginal bacterial composition among different community state type (CST) groups at species level. (a) The heatmap of vaginal bacterial composition in each CST group. The shading represents the level of relative abundance (RAB) of a given taxon. (b) The cladogram of vaginal bacterial taxa in different CST groups. The size of yellow dots shows the read rank of each species, and the transparency of colored squares around each internal circle indicates its RAB level. The bars outside the circle represent the original reads of the corresponding taxon on a log_10_ scale.

The participants with available 16S rRNA data (*n* = 359) were clustered into five community state type (CST) groups and termed CST-I (134, 37.3%), CST-II (13, 3.6%), CST-IIIa (107, 29.8%), CST-IIIb (45, 12.5%), and CST-IV (60, 16.7%), respectively (Fig. 1). CST-I was the most prevalent group, dominated by Lactobacillus crispatus, and then followed by CST-IIIa, which contained a high abundance of a single Lactobacillus iners. CST-IIIb was also dominated by Lactobacillus iners but had a mixture with Gardnerella vaginalis, Lactobacillus crispatus, and other non-*Lactobacillus* spp. CST-II was the group with the lowest prevalence and was primarily dominated by Lactobacillus paragasseri*/gasseri*. CST-IV had the most diverse bacterial communities among all the groups and was mostly characterized by Gardnerella vaginalis and Atopobium vaginae. Between-group comparisons suggested that the CST groups had a slight difference in baseline gestational age and first-time pregnancy status after pairwise comparisons and multiple corrections (*q *< 0.05; see Table S2 and Fig. S7 at https://doi.org/10.5281/zenodo.5054434). The majority of participants with available microscopic examinations did not show evidence of vaginitis, as suggested by white blood cell counts and *Trichomonas* and yeast tests (Table S3 at https://doi.org/10.5281/zenodo.5054434).

The 16S rRNA read summary results at the genus level suggested *Lactobacillus* topped all the 16S reads in this study, next followed by *Gardnerella*, *Atopobium*, *Prevotella*, *Dialister*, *Megasphaera*, and *Shuttleworthia* (Table S4 at https://doi.org/10.5281/zenodo.5054434). Most *Lactobacillus* reads were comprised of Lactobacillus crispatus and Lactobacillus iners (Table S4 at https://doi.org/10.5281/zenodo.5054434). The genus-level correlation network showed that *Lactobacillus* was negatively correlated with *Prevotella*, *Atopobium*, *Dialister*, and *Gardnerella*, whereas the species-level correlation network suggested that positive relationships could exist within most of the *Lactobacillus* species members (Fig. S8 at https://doi.org/10.5281/zenodo.5054434).

### Structural correlations between host genetic variations and individual VBTs.

The estimation of narrow-sense genetic heritability for individual VBTs did not show any statistical estimate with respect to the alpha diversity metrics, the beta diversity metrics, or the binary or continuous traits of vaginal bacterial taxa (*P* > 0.05; Tables S6 and S7 at https://doi.org/10.5281/zenodo.5054434). However, there were seven 16S extrapolated MetaCyc pathways that showed a statistically significant heritability when estimated with either single nucleotide polymorphisms (SNPs) or indels, including PWY-5005, PWY-6396, 1CMET2-PWY, FUC-RHAMCAT-PWY, KDO-NAGLIPASYN-PWY, METH-ACETATE-PWY, and PWY-7315 (*P* < 0.05; Tables S6 and S7 at https://doi.org/10.5281/zenodo.5054434). Among these pathways, 1CMET2-PWY (N10-formyl-tetrahydrofolate biosynthesis) was a prevalent MetaCyc pathway among the participants with available 16S amplicon data (Table S4 at https://doi.org/10.5281/zenodo.5054434). The fixation index (FST) was also estimated to identify whether the SNPs in the population were differentiated with respect to each binary VBT. However, all the estimated FST values were quite close to zero, suggesting a high homogeneity in the genetic background of the study participants (Table S8 at https://doi.org/10.5281/zenodo.5054434).

The principal-component (PC) structural correlations suggested several SNP-based genomic PCs were significantly associated with Faith phylogenetic diversity (PD) and six MetaCyc pathways including PHOSLIPSYN-PWY, PWY-5005, PWY-621, PWY-6317, PWY4FS-8, and THRESYN-PWY, while the indel-based genomic PCs were associated with the presence/absence (P/A) of *Actinobacteriota* [Phylum], the P/A and relative abundance (RAB) of *Proteobacteria* [Phylum], the dominance/nondominance (D/ND) of Lactobacillus iners [Species], and 17 MetaCyc pathways after the false-discovery rate (FDR) multiple corrections (*q *< 0.05; Tables S9 and S10 at https://doi.org/10.5281/zenodo.5054434). The MetaCyc pathway PWY-6317 (galactose degradation I [Leloir pathway]) had a significant association with both SNP and indel-based PCs (*q *< 0.05). The copy number variation (CNV)-based genomic PCs were significantly associated with the RAB of Atopobium vaginae [Species] and 36 MetaCyc pathways (*q *< 0.05; Table S11 at https://doi.org/10.5281/zenodo.5054434).

### Genome-wide association analysis between genetic variants and VBTs.

Totally, a sum of 72 host genetic variant (SNP, indel, or CNV)-VBT associations were found that reached the genome-wide significance level (*P* < 5 × 10^−8^), and 3,415 variant-VBT associations reached the suggestive significance level (*P* < 1 × 10^−5^) with a genomic inflation factor (GIF) *λ* of <1.1 (Tables S12, S13, and S14 at https://doi.org/10.5281/zenodo.5054434). The Manhattan plots for the individual associations between genetic variants and VBTs were archived in the supplemental materials (Doc S4, Doc S5, and Doc S6 at https://doi.org/10.5281/zenodo.5054434).

**FIG 2:**
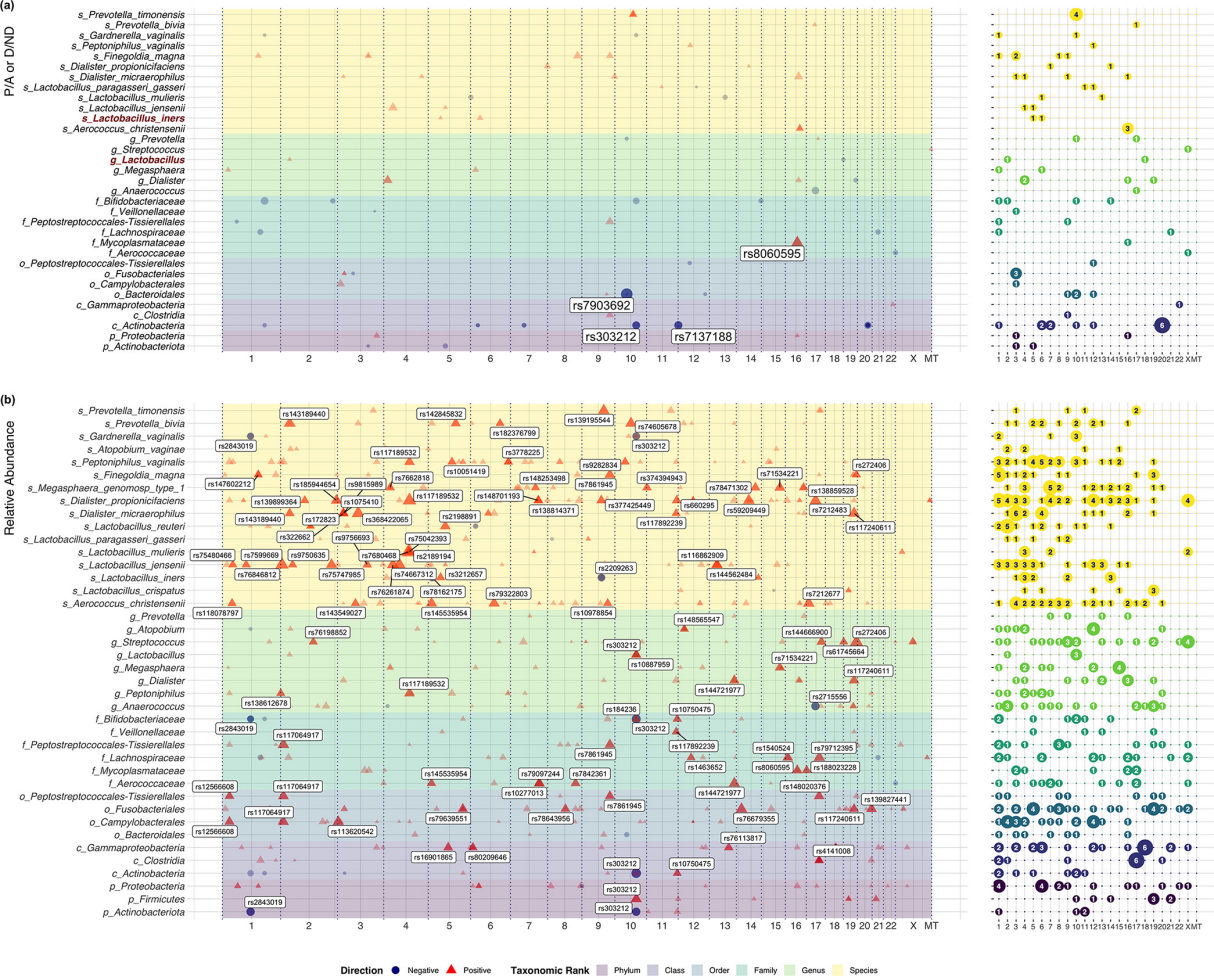
The association between single genetic polymorphisms (SNPs) and the vaginal bacterial taxa in pregnant women (λ < 1.1). All the SNPs that reached the suggestive significance level (*P* < 1 × 10^−5^) were shown in the plots, but only those with *P* values lower than 1 × 10^−6^ were highlighted with a relatively low transparency in filled colors and annotated with variant labels. The left panel shows the significant SNPs (*P* < 1 × 10^−5^) and their corresponding positions on different chromosomes, whereas the right panel shows the number of suggestively significant markers on each chromosome accordingly. The background color represents different taxonomic levels of each bacterial taxon. Covariates including the first-time pregnancy status, baseline GA, and 10 SNP-PCs were adjusted. (a) The associations between host SNPs and the binary VBTs including P/A and D/ND of a taxon given its distribution in study samples. Text in bold red indicates that the dominance of the taxon was studied, whereas the P/A trait was studied for the remaining taxa. (b) The associations between host SNPs and the RAB of vaginal bacterial taxa.

**FIG 3:**
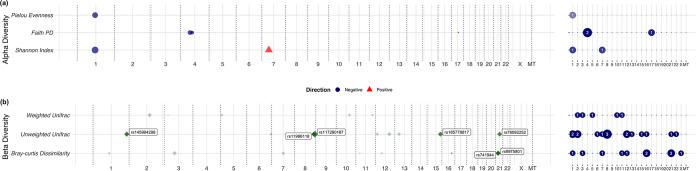
The association between single genetic polymorphisms (SNPs) and the bacterial diversity metrics in pregnant women (λ < 1.1). All the SNPs that reached the suggestive significance level (*P* < 1 × 10^−5^) were shown in the plots, but only those with *P* values lower than 1 × 10^−6^ were highlighted with a relatively low transparency in filled colors and annotated with variant labels. The left panel shows the significant SNPs (*P* < 1 × 10^−5^) and their corresponding positions on different chromosomes, whereas the right panel shows the number of suggestively significant markers on each chromosome accordingly. Covariates including the first-time pregnancy status, baseline GA, and 10 SNP-PCs were adjusted. (a) The associations between host SNPs and the alpha diversity metrics. (b) The associations between host SNPs and the beta diversity metrics by MANOVA.

For the SNPs, the single variant association analyses identified 68 SNP-VBT associations that reached a genome-wide significance level (*P* < 5 × 10^−8^). Regional assessments for these SNPs by locuszoom based on the imputed 1000 Genomes Project reference panel data within a ±250-kb slide window were archived in the supplemental materials (Doc S1 at https://doi.org/10.5281/zenodo.5054434). These SNP-associated VBTs included Unweighted UniFrac, the P/A of *Bacteroidales* [Order], the RAB of *Actinobacteria* [Class], *Bifidobacteriaceae* [Family], Streptococcus [Genus], Dialister micraerophilus [Species], Dialister propionicifaciens [Species], Lactobacillus jensenii [Species], Lactobacillus mulieris [Species], and 27 extrapolated MetaCyc pathways (*P* < 5 × 10^−8^). Among these SNPs that had a genome-wide significant association with the VBTs, only seven SNPs had a minor allele frequency (MAF) of >0.05 including rs7903692, rs7842439, rs10806401, rs13004173, rs11831423, rs303212, and rs75042393. Specifically, rs7903692 located at the DRGX gene on chromosome 10 had a genome-wide significant association with the P/A of *Bacteroidales* [Order] (odds ratio [OR] = 0.35; 95% confidence interval [95% CI], 0.24 to 0.50; *P* = 1.27 × 10^−8^). rs7842439, rs10806401, rs13004173, and rs11831423 showed a significant association with one of the MetaCyc pathways including LEU-DEG2-PWY, PWY-5747, PWY0-321, and TYRFUMCAT-PWY, respectively (*P* < 5 × 10^−8^). However, these four MetaCyc pathways shared a low abundance and prevalence (<60%) in the study participants with available 16S amplicon data. rs75042393 located 225,257 bp away from the DKK2 gene on chromosome 4 had a genome-wide significant association with the RAB of *Lactobacillus mulieris* [Species]. The T allele of rs303212 located at the IFIT1 gene on chromosome 10 had a negative association with the RAB of *Actinobacteria* [Class] (*P* = 1.64 × 10^−8^) and *Bifidobacteriaceae* [Family] (*P* = 4.48 × 10^−8^). These two bacterial taxa were the higher-level taxa of Gardnerella vaginalis [Species]. Besides, rs303212 also had a suggestive association with the P/A of *Actinobacteria* [Class] (*P* = 9.49 × 10^−7^), *Bifidobacteriaceae* [Family] (*P* = 2.32 × 10^−6^), Gardnerella vaginalis [Species] (*P* = 7.85 × 10^−6^), the RAB of *Actinobacteriota* [Phylum] (*P* = 1.22 × 10^−7^), *Firmicutes* [Phylum] (*P* = 1.18 × 10^−7^), *Lactobacillus* [Genus] (*P* = 4.62 × 10^−7^), Gardnerella vaginalis [Species] (*P* = 3.11 × 10^−7^), and the beta diversity Weighted UniFrac (*P* = 5.54 × 10^−6^), where *Firmicutes* [Phylum] is the higher-level taxon of *Lactobacillus* [Genus]. The partial allele effect of rs303212 was negatively correlated with the RAB of *Bifidobacteriaceae* but positively correlated with *Lactobacillus* (see Fig. 5a and b). Genes near this SNP (within ±250 kb) included CH25H, LIPA, IFIT2, IFIT3, IFIT1B, IFIT1, IFIT5, SLC16A12-AS1, SLC16A12, PANK1, and MIR107 as suggested by the locuszoom plot with the imputed 1000 Genomes Project reference panel data (see Fig. 5c). For the most common genus-level bacterial taxon, *Lactobacillus* [Genus], two SNPs including rs4670222 and rs62096874, which are located at LINC00211 and NFATC1, respectively, were identified that had a suggestive association with its dominance status and four SNPs including rs4670222, rs303212, rs184236, and rs10887959 had a suggestive association with its relative abundance (*P* < 1 × 10^−5^). As Gardnerella vaginalis [Species] is the only species of *Gardnerella* [Genus], it made up the majority of *Bifidobacteriaceae* [Family] in women’s vaginas in this study. In this study, a total of six SNPs showed a suggestive association with the RAB of Gardnerella vaginalis [Species], including rs2843019 located at HMGCS2 (*P* = 7.57 × 10^−7^), rs2208692 located at HMCN1 (*P* = 8.67 × 10^−6^), rs138140882 located at CYCS (*P* = 4.01 × 10^−6^), rs150145959 located 392,908 bp away from MIR3924 (*P* = 5.97 × 10^−6^), rs303212 located at IFIF1 (*P* = 3.11 × 10^−7^), and rs184236 located at IFIT5 (*P* = 4.66 × 10^−6^). There were no SNPs that showed a genome-wide association with the two most prevalent *Lactobacillus* spp. (Lactobacillus iners and Lactobacillus crispatus) (*P* > 5 × 10^−8^); however, several SNP-specific associations with the P/A of Lactobacillus iners [Species], the RAB of Lactobacillus iners [Species], and the RAB of Lactobacillus crispatus [Species] reached the suggestive significance level (*P* < 1 × 10^−5^). All the SNPs that showed a suggestive association (*P* < 1 × 10^−5^) with the vaginal bacterial taxa and the diversity metrics were summarized in Fig. 2 and 3, respectively.

The analyses on the associations between indels and VBTs (Table S13 at https://doi.org/10.5281/zenodo.5054434) identified only the C deletion in rs201053982 at the KATNAL2 gene on chromosome 18 as having a genome-wide significant association with P105-PWY (*P* = 4.37 × 10^−8^). rs201053982 also had a suggestive association with some other MetaCyc pathways and the RAB of *Gammaproteobacteria* [Class] (*P* = 5.11 × 10^−7^). Additionally, the AT deletion in rs146695045 at the ZNF28 gene had a suggestive association with the P/A and RAB of *Fusobacteriales* [Order] (*P* = 9.46 × 10^−6^ and 2.67 × 10^−6^, respectively). These indels did not show any suggestive significant association with the alpha or beta diversity metrics (*P* > 1 × 10^−5^).

**FIG 4:**
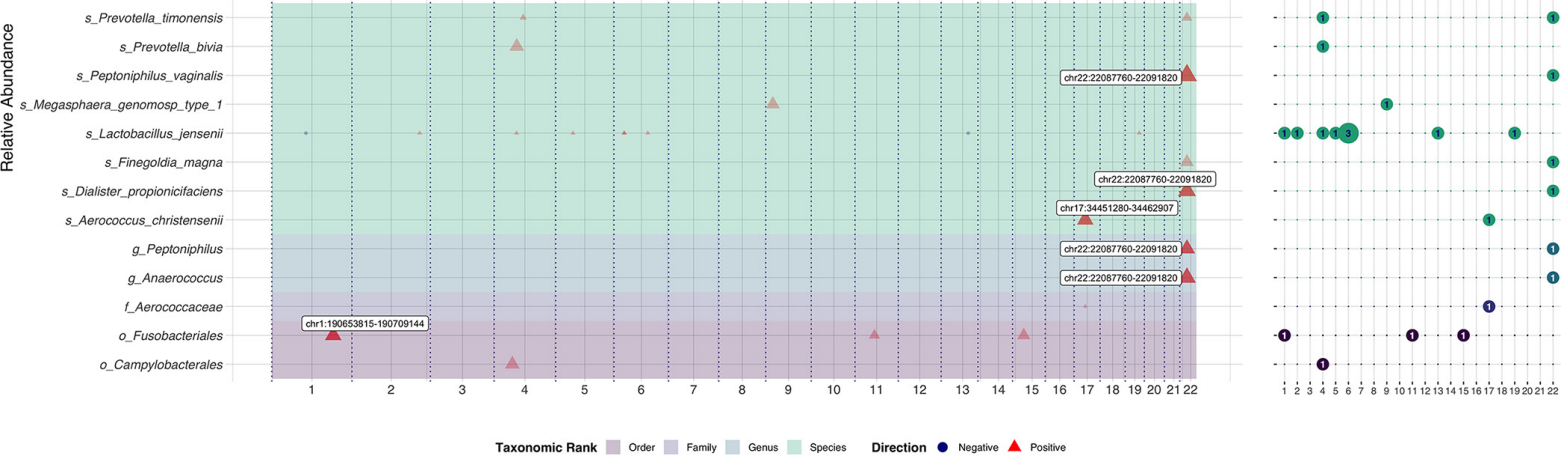
The association between copy number variations (CNVs) and the RAB of vaginal bacterial taxa in pregnant women (λ < 1.1). All the CNVs that reached the suggestive significance level (*P* < 1 × 10^−5^) were shown in the plots, but only those with *P* values lower than 1 × 10^−6^ were highlighted with a relatively low transparency in filled colors and annotated with variant labels. The left panel shows the significant SNPs (*P* < 1 × 10^−5^) and their corresponding positions (center of CNV) on different chromosomes, whereas the right panel shows the number of suggestively significant markers on each chromosome accordingly. The background color represents different taxonomic levels of each bacterial taxon. Covariates including the first-time pregnancy status, baseline GA, and 10 CNV-PCs were adjusted.

The CNVs showed only a suggestive association with the MetaCyc pathways and the RAB of specific bacterial taxa (*λ* < 1.1 and *P* < 1 × 10^−5^; Fig. 4 and also Table S14 at https://doi.org/10.5281/zenodo.5054434). Among these associations, only the associations between CNV chr4:161865780-161929246 and low-abundant pathways including PWY-5180, PWY-5182, and PWY-5415 reached the genome-wide significance level (*P* < 5 × 10^−8^).

### Joint analyses for multiple VBTs.

Joint association analyses for the RAB of multiple species-level taxa were performed to investigate the potential pleiotropy of the genetic variants. The results showed that none of the CNVs or indels had a suggestive association with all the 16 or the top three prevalent species-level bacterial taxa. However, a total of 20 SNPs located across the human genome were identified that had a suggestive association with the RAB of the top three species-level bacterial taxa Lactobacillus crispatus, Lactobacillus iners, and Gardnerella vaginalis (Table S15 at https://doi.org/10.5281/zenodo.5054434). These three taxa accounted for over 80% of all the bacterial reads in the participants with available 16S rRNA data. Among these SNPs, rs2843019, rs303212, and rs184236 had the highest MAF among the SNPs identified, which were 0.486, 0.394, and 0.438 and located at the HMGCS2, IFIT1, and IFIT5 genes, respectively. There were some other SNPs that also had a suggestive association with all the 16 species-level bacterial taxa. However, the estimated *λ* for genomic inflation suggested the results were inflated (*λ* = 1.108; Table S16 at https://doi.org/10.5281/zenodo.5054434).

### Gene mapping and enrichment analyses for all the mapped genes and individual VBTs.

The GTEx expression quantitative trait locus (eQTL) mapping identified a total of 882 eQTL-related genes that were expressed in 47 tissues for the SNPs that reached the suggestive significance level (Table S17 at https://doi.org/10.5281/zenodo.5054434) and one gene in three tissues for indels (Table S18 at https://doi.org/10.5281/zenodo.5054434). The vagina-specific eQTL-related genes included NBPF26, RP11-92G12.3, MIR6891, DOPEY1, RP11-274B21.13, CICP14, RP11-274B21.10, CPSF1, IFIT5, IFIT1, AL163953.3, JMJD7, SLC28A2, CTD-2651B20.3, CTD-2651B20.4, PRMT7, CHRNE, ZNF600, and CD40. Gene-based association analysis for SNPs identified 340 unique genes that reached a suggestive significance level after multiple corrections (*λ* < 1.1, *P* < 1 × 10^−5^, and *q* < 0.05; Table S19 at https://doi.org/10.5281/zenodo.5054434), whereas the association analysis for indels identified six (ZNF600, PABPN1P2, ZNF28, KATNAL2, ELOA3, and ELOA2) accordingly (Table S20 at https://doi.org/10.5281/zenodo.5054434).

Finally, a total of 1,145 unique mapped genes with available Entrez ID (Table S21 at https://doi.org/10.5281/zenodo.5054434), which were related to at least one VBT (the diversity metrics, the bacterial taxa, or MetaCyc pathways), were submitted to FUMA GENE2FUNC for overall enrichment analysis (Doc S2 at https://doi.org/10.5281/zenodo.5054434). The differentially expressed gene (DEG) sets for these genes were found to have upregulated expression in tissues like Skin Sun Exposed Lower Leg, Esophagus Mucosa, Vagina, Skin not Sun Exposed Suprapubic, Artery Coronary, Lung, Adipose Subcutaneous, Nerve Tibial, and Esophagus Gastroesophageal Junction (*P*_bonferroni_ < 0.05; Doc S2 p5-6 at https://doi.org/10.5281/zenodo.5054434). Joint gene set clustering suggested that these genes were involved with multiple biological pathways like metal ion homeostasis, host immunity, lipid metabolism, hormone signaling, and so forth, given 19 different databases (Table S22 and Doc S2 at https://doi.org/10.5281/zenodo.5054434). For instance, the Reactome database identified six pathways including *REACTOME METALLOTHIONEINS BIND METALS*, *REACTOME SYNTHESIS OF LEUKOTRIENES LT AND EOXINS EX*, *REACTOME RESPONSE TO METAL IONS*, *REACTOME PHOSPHOLIPID METABOLISM*, *REACTOME GLYCEROPHOSPHOLIPID BIOSYNTHESIS*, and *REACTOME INNATE IMMUNE SYSTEM*, whereas the Wikipathways database identified three pathways including *Zinc homeostasis*, *Allograft Rejection*, and *Oxidation by Cytochrome P450* (*q *< 0.05).

Enrichment analysis results for individual VBTs were shown in Table S23 at https://doi.org/10.5281/zenodo.5054434. Among these gene sets, seven KEGG pathways (including *hsa00072∼Synthesis and degradation of ketone bodies*, *hsa00260∼Glycine*, *serine and threonine metabolism*, *hsa00280∼Valine*, *leucine and isoleucine degradation*, *hsa00650∼Butanoate metabolism*, *hsa00900∼Terpenoid backbone biosynthesis*, *hsa01230∼Biosynthesis of amino acids*, and *hsa03320∼PPAR signaling pathway*) and four Reactome pathways including *Amino acid synthesis and interconversion (transamination)*, *Ketone body metabolism*, *Serine biosynthesis*, and *Synthesis of Ketone Bodies* were significantly enriched for the RAB of Gardnerella vaginalis [Species] (*q *< 0.05). Except for *hsa01230∼Biosynthesis of amino acids*, *hsa03320∼PPAR signaling pathway*, and *Amino acid synthesis and interconversion* (*transamination*), these pathways were also significantly enriched for the RAB of *Bifidobacteriaceae*. For *Lactobacillus*, there were one KEGG pathway (*hsa05168∼Herpes simplex infection*) and two Reactome pathways (*Interferon Signaling* and *Interferon alpha/beta signaling*) significantly enriched for its relative abundance (*q *< 0.05). The Reactome pathways *Interferon Signaling* and *Interferon alpha/beta signaling*, which were generally involved with IFIT gene families like IFIT1 and IFIT2, were also related to the MetaCyc pathways like ARO-PWY [chorismate biosynthesis I], COMPLETE-ARO-PWY [superpathway of aromatic amino acid biosynthesis], NONOXIPENT-PWY [pentose phosphate pathway (nonoxidative branch)], PANTO-PWY [phosphopantothenate biosynthesis I], PWY-5121 [superpathway of geranylgeranyl diphosphate biosynthesis II (via MEP)], PWY-6163 [chorismate biosynthesis from 3-dehydroquinate], PWY-6895 [superpathway of thiamine diphosphate biosynthesis II], PWY-6897 [thiamine salvage II], PWY-7323 [superpathway of GDP-mannose-derived O-antigen building blocks biosynthesis], and PWY-7560 [methylerythritol phosphate pathway II] (*q *< 0.05). Most of these MetaCyc pathways shared a relatively high abundance and prevalence in the study samples.

## DISCUSSION

**FIG 5:**
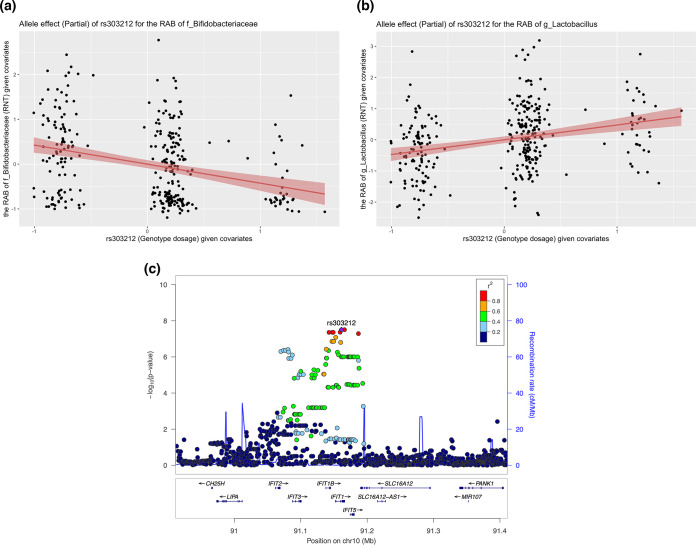
The associations between rs303212 and common bacterial taxa (*Bifidobacteriaceae* and *Lactobacillus*). (a) The partial allele effect of rs303212 on the RAB of *Bifidobacteriaceae* given adjusted covariates including first-time pregnancy status, baseline gestational age, and 10 SNP-PCs. (b) The partial allele effect of rs303212 on the RAB of *Lactobacillus* given adjusted covariates including first-time pregnancy status, baseline gestational age, and 10 SNP-PCs. (c) locuszoom plot for rs303212 with respect to the RAB of *Bifidobacteriaceae* within a ±250-kb window using the imputed 1000 Genomes Project phase 3 reference panel data.

In this study, a total of 72 host genetic variant (SNPs, indels, or CNVs)-VBT associations were found that reached the genome-wide significance level (*P* < 5 × 10^−8^), and 3,415 associations reached the suggestive significance level (*P* < 1 × 10^−5^) with a GIF *λ* of <1.1. The majority of these SNPs that reached the genome-wide significance level had a relatively low MAF, and only seven of them had MAFs greater than 0.05, including rs7903692, rs303212 on chromosome 10, rs7842439 on chromosome 8, rs10806401 on chromosome 6, rs13004173 on chromosome 2, rs11831423 on chromosome 12, and rs75042393 on chromosome 4. Of these variants, the SNP rs303212 located at the IFIT1 gene was the most eye-catching variant and had a relatively high MAF in the study samples (Fig. 5). This SNP not only had a genome-wide significant association with the RAB of *Actinobacteria* [Class] and *Bifidobacteriaceae* [Family] (*P* < 5 × 10^−8^), which are the higher-level taxa of Gardnerella vaginalis, but also had a suggestive association with the RAB of other common vaginal bacterial taxa including *Actinobacteriota* [Phylum], *Firmicutes* [Phylum], *Lactobacillus* [Genus], and Gardnerella vaginalis [Species] (*P* < 1 × 10^−5^). Another SNP that had a relatively high MAF and a genome-wide significant association with the bacterial taxa was rs7903692. The T allele of rs7903692 located at DRGX was negatively associated with the P/A of *Bacteroidales* [Order]. rs75042393 (225,257 bp to DKK2) was significantly associated with the RAB of *Lactobacillus mulieris*, which was a recently identified Gram-positive, nonmotile, non-spore-forming, catalase-negative, coccobacillus-shaped bacterium (25, 26). The remaining SNPs with a MAF greater than 0.05 had a genome-wide association with the MetaCyc pathways which were not abundant and prevalent in the samples with available 16S amplicon data. During pregnancy, the vaginal bacteriome is generally characterized by dominant *Lactobacillus*, which accounted for the majority of all the sequenced reads in this study. Only two SNPs including rs4670222 and rs62096874, which are located at LINC00211 and NFATC1, respectively, were identified that had a suggestive association with its dominance status. These findings suggest that the overall structure of the vaginal bacteriome in pregnancy may not be simply influenced by a small fraction of genetic variants but by a broad range of variants across the host genome. Joint analyses for multiple traits identified few SNPs associated with the RAB of the top three species-level bacterial taxa (Lactobacillus crispatus, Lactobacillus iners, and Gardnerella vaginalis) at a suggestive significance level with a subtle population structure (*λ* < 1.1), suggesting the potential pleiotropy of several SNPs on the colonization of vaginal bacterial communities.

An increase in the RAB of one taxon often brings a decrease in one another. Unlike the bacteria colonizing other human body sites, the polarization of bacterial communities is more common in the vagina, as it is often colonized by a few dominant bacteria, especially in pregnant women (18, 19, 27, 28). The bacterial taxa *Actinobacteria* and *Bifidobacteriaceae* are on the same lineage and are the higher-level taxa of Gardnerella vaginalis. As Gardnerella vaginalis made up the majority of the reads of *Bifidobacteriaceae* and its genus-level taxon *Gardnerella* was negatively associated with the most common genus-level taxon *Lactobacillus*, rs303212 and some adjacent SNPs like rs184236 and rs10887959 were also identified that had either a suggestive or genome-wide association with the RAB of Gardnerella vaginalis, *Lactobacillus*, and their higher-level taxa in this study. For instance, the T allele of rs303212 showed a positive association with the RAB of *Lactobacillus* but had a negative association with the RAB of Gardnerella vaginalis, which often competes with the *Lactobacillus* spp. for an ecological niche in the female lower genital tract (29, 30). As the major lower-level bacterial taxon of *Bifidobacteriaceae*, Gardnerella vaginalis was also often reported as a potentially risky bacterium for women's reproductive health. It can form a specific biofilm on vaginal epithelium and recruit other bacteria seen in BV, leading to a polymicrobial environment in women’s vaginas (31). Therefore, the associations between rs303212 and the RAB of primary vaginal bacterial taxa identified may be possibly driven by the negative correlation between Gardnerella vaginalis and *Lactobacillus* spp.

rs303212 and its adjacent SNPs are located at or near other IFIT gene family members like IFIT1 and IFIT5, which belong to the interferon (IFN)-stimulated genes (ISGs). The human IFIT gene family is generally comprised of four members (IFIT1, IFIT2, IFIT3, and IFIT5) and plays a pivotal role in host antiviral defense (32, 33). This gene family encode the IFN-induced proteins with tetratricopeptide repeats and can be strongly induced by several IFNs like type I IFNs and type III IFNs (34–36). The relationships between the IFIT gene family and the colonization by vaginal bacteria were not frequently reported in previous studies or in the GIMICA platform (37) (Table S23 at https://doi.org/10.5281/zenodo.5054434). However, recently a study has found that the lactic acid bacteria, which are generally considered beneficial bacteria in the vagina, can activate type I interferon production and induce IFIT1 activation via the intracellular cytosolic sensor STING (38). The *interferon signaling* or *interferon alpha/beta signaling* pathways were also enriched for a variety of VBTs in this study like the P/A of *Actinobacteria* [Class] and the RAB of *Actinobacteriota* [Phylum] and *Lactobacillus* [Genus], suggesting a potential effect of interferon signaling on the colonization by these vaginal bacteria. These findings may support the point that IFIT1 may function as a modulator in influencing the susceptibility to bacteria through the interferon response (39). Additionally, *Interferon Signaling* and *Interferon alpha/beta signaling* were also enriched for some abundant MetaCyc pathways, indicating that the interferon-related signaling might also play an important role in the vaginal bacterial community functions.

Generally, bacterial associations with host genetic variants are considered to be body site specific. However, the associated variants of the vaginal bacteriome were not as widely considered as those of the gut bacteriome (14, 18–20). The immune-related gene-targeted association studies have revealed the potential influence of polymorphisms of genes such as TLR4 and interleukin-1 receptor antagonist on the colonization by specific pathogenic bacteria in the vagina (Table S23 at https://doi.org/10.5281/zenodo.5054434), but an early genomic study based on whole-genome sequencing with 80 vaginal-posterior fornix samples identified only a few taxon-related single nucleotide variants after a stringent Bonferroni correction (20, 22, 40–43). These studies provided some evidence regarding the potential influences of host genetic factors on vaginal bacterial communities.

Another related GWAS was recently reported by Mehta et al. (21), where the association between host SNPs and the vaginal bacterial composition was investigated in Kenyan women. Several SNPs have been identified that have a suggestive significant association with the RAB of Lactobacillus crispatus, Lactobacillus iners, and Gardnerella vaginalis and the Shannon index. These suggestively significant associations were not replicated in this study. However, the association between rs527430 and the RAB of Lactobacillus iners shared the same direction as what was found in the Kenyan GWAS though the *P* value was not statistically significant in this study. Several reasons might account for the differences. First, the Kenya GWAS recruited 171 women of reproductive age with a high proportion of participants being infected with herpes simplex virus or HIV, whereas women in pregnancy were the primary interest in this study. Second, both of the studies excluded many SNPs during the quality control (QC) process due to the small sample sizes, possibly leading to relatively sparse and divergent markers being captured in the association analysis. Last, the genetic background of the study populations and the designed chip loci might also result in the different genetic variants remaining to be tested. Despite these differences, other similarities were also found regarding the genes mapped and identified biological pathways. For instance, the GO term *GO:0001106∼RNA polymerase II transcription corepressor activity* was also enriched for Shannon index in this study. Additionally, despite that the results were not always statistically significant, the G-protein-coupled receptor (GPCR)-related pathways were also clustered by different genes with respect to multiple VBTs including the P/A of *Bifidobacteriaceae*, which is the higher-level taxon of Gardnerella vaginalis, whereas it was identified for the major vaginal bacteria like Lactobacillus crispatus and Gardnerella vaginalis in the Kenyan GWAS.

The immunosuppressed host-graft model, which regards the immune cells at the maternal-fetal interface as an immune response to the semiallogeneic fetus, was once considered the paradigm in the immunology of pregnancy. However, a recent review suggested that the response instead begins with a proinflammatory stage to facilitate blastocyst implantation, then shifts to the anti-inflammatory stage to allow fetal growth, and finally shifts back to the proinflammatory stage for labor and delivery (9). As most study participants were enrolled at the early second trimester, immune-related genetic associations may mostly represent an anti-inflammatory stage of the immune response to the maternal vaginal bacteria. Furthermore, trophoblast cells that attach the zygote to the uterine wall can express TLRs and are responsible for sensing and responding to bacterial products (9). The response to TLR4 ligation by lipopolysaccharide (LPS) can also induce the production of type I interferons that are involved in the modulation of inflammatory responses via their effects on TLR signaling pathways (39, 44, 45). Although a direct single-marker association on TLR4 polymorphisms was not captured (no SNP that passed QC was located at this gene in this study), the polymorphisms of other ISGs like the IFIT gene family may be involved in the modulation of the TLR4 signaling pathway by LPS and act as a regulator of both proinflammatory genes and interferon-stimulated genes so as to alter the susceptibility of bacteria as shown by previous evidence (39). In addition, genes like ATF1 and S100A9 were also enriched in some TLR-related pathways for the P/A of Peptoniphilus vaginalis and the RAB of Finegoldia magna, suggesting their potential role in influencing the colonization by these two bacteria. Previous evidence also showed the functionality of these genes in influencing the response to the vaginal infections by pathogens. For instance, S100A9 alarmins can be produced from the vaginal epithelium in response to *Candida* infection (46). These findings reiterated the functional roles of human immunology in shaping the vaginal bacteriome.

In addition, similar to the heritability estimation for the transmission of the gut bacteriome, a relatively high heritability estimate with statistical significance for the vaginal bacterial taxa was not detected in this study. Besides the lack of statistical power caused by the small sample size, a few other reasons might explain the lack of statistical significance. The statistical narrow-sense heritability defines the proportion of phenotypic variation in a population that is explained by genetic variation (47). However, the estimates can be affected by strong purifying selection, environmental noise, and nonadditive genetic variance (16). As only relatively healthy pregnant women at a single site were enrolled, a strong purifying selection was carried out in this study, possibly leading to a fixation of genetic variants and a reduction in the heritability estimates. Moreover, as suggested by previous population genetic theories and empirical studies, heritable components of the microbiota are more likely to be involved in genetic adaptations, and the associated fitness may not provide the relative contributions from genetic and environmental variance to a trait, which may also lower the heritability estimation (48–52). Suzuki and Ley also demonstrated that the heritable components of the microbiota in the host population could be fixed long term for genetic adaptation to replace or recruit the microbes with beneficial effects on hosts (16). Therefore, heritable genetic variants in this study may provide merely a starting point to identify the microbes that are undergoing host genetic adaptations to the vaginal environment in pregnancy. Nonetheless, several MetaCyc pathways had statistically significant heritability estimates, suggesting the potential heritability of microbial functionalities in adaptation to the selection by the host-environment interactions, although most of these MetaCyc pathways were not abundant in this study.

In summary, this study adds to a limited body of knowledge on the association between host genetic variants and the vaginal bacteriome in pregnancy. However, as clinical measurements regarding sexual transmitted diseases (STDs) were involved with too many missing values, a thorough assessment of infection status for the participants was not applicable in this study, which might weaken the comparability of the results from other studies which assessed the STDs. Additionally, as a sample of pregnant Chinese women with a relatively high homogeneity in genetic background and demographics was studied, the results from the association and enrichment analyses may underscore the variants with high specificity. Nevertheless, the power of the study results may still be subject to the small sample size. Further studies with larger sample sizes are still preferable to confirm these associations. Understanding the genetic background of the vaginal bacteriome in pregnancy may disclose the innate factors that function to guarantee a favorable pregnancy outcome so as to facilitate the establishment of specific strategies for prevention and clinical treatment of pregnancy complications.

### Conclusion.

The vaginal bacteriome may be influenced by a number of genetic variants across the host genome. The polymorphisms in the IFIT gene family and the related interferon signaling pathway may be important in determining the colonization by the common vaginal bacteria during pregnancy.

## MATERIALS AND METHODS

### Study population and sampling.

This study was conducted among a cohort of pregnant women from Anqing Municipal Hospital in Anhui Province, China. During their prenatal visits, pregnant women who gave informed consent and who met the following criteria were included: (i) aged 18 or older, (ii) baseline gestational age (GA) in the first or second trimester, (iii) having not taken any antibiotics in the previous 4 weeks, and (iv) absent of serious organic or systemic diseases (like coronary heart disease, stroke, leukemia, and so forth). Initially, a total of 1,561 pregnant women were enrolled from 22 February 2018 to 22 January 2020 prior to the COVID-19 pandemic and were followed up for their pregnancy outcomes. Then, a sample of 390 participants was drawn from the cohort baseline population using a systematic random sampling method with a sampling ratio of 1:4. After excluding those who (i) had missing or unqualified vaginal samples for 16S rRNA gene amplicon sequencing (*n* = 13), (ii) had sexual intercourse in the previous 48 h (*n* = 14), (iii) had multiple pregnancies (*n* = 3), or (iv) had reported inconsistent key data between the baseline and follow-up in medical records (*n* = 1), a final sample of 315 out of 359 eligible participants with the 16S rRNA data had corresponding SNP array data for the association analysis between host genetic variants and VBTs (see Fig. S1 at https://doi.org/10.5281/zenodo.5054434). The participants with both host genome and vaginal bacteriome data had a median age of 28 years (interquartile range [IQR], 25 to 30). Most pregnant women were enrolled during their second trimester with a median 16.4 weeks of pregnancy (IQR, 15.7 to 17.0). These participants did not exhibit significant differences in age, baseline GA, age at menarche, prepregnancy body mass index, first-time pregnancy (primigravida or not), smoking, drinking habits, colporrhagia within 2 weeks, diarrhea or constipation within 2 weeks, vaginal douching habit, family history of hypertension and diabetes, or self-reported gestational diabetes at baseline compared with the original cohort participants (*P* > 0.05; Table S5 at https://doi.org/10.5281/zenodo.5054434). The study protocol was reviewed and approved by the ethical committee of the School of Public Health, Fudan University (IRB#2017-09-0636).

### Sample collection.

At baseline, midvaginal samples were drawn by experienced obstetricians using sterile swabs. The vagina was scraped with the swab in a clockwise rotation for five spins. Paired swabs were collected for each subject. One swab was placed in a collection tube with preservation solution and stored in a low-temperature freezer at −80°C before genome sequencing, and the other was inspected by wet mount for clinical purposes. A 5.0-ml blood sample was taken from each participant by the hospital laboratory staff and also stored at −80°C before genotyping.

### DNA extraction, 16S rRNA amplicon sequencing, and host DNA genotyping.

The vaginal bacterial genomic DNA was extracted from frozen swabs using a mixture of cetyltrimethylammonium bromide and sodium dodecyl sulfate methods. The V3-V4 hypervariable regions of 16S rRNA genes were amplified with specific barcodes on the primers for PCR and then sequenced on an Ion S5 XL instrument (Novogene Co., Ltd., Beijing, China). The primers were as follows: 341F, 5′-CCTAYGGGRBGCASCAG-3′, and 806R, 5′-GGACTACNNGGGTATCTAAT-3′. PCR amplifications were conducted in a final reaction volume of 30 μl mixture containing 15 μl of 2× Phusion Master Mix, 3 μl of primer (2 μM), 10 μl of the genomic DNA (gDNA) (1 ng/μl), and 2 μl H_2_O. The reaction consisted of one cycle of initial denaturation at 98°C for 1 min, followed by 30 cycles at 98°C for 10 s, annealing at 50°C for 30 s, and extension at 72°C for 30 s, and a final cycle at 72°C for 5 min. The GeneJET gel extraction kit (Thermo Fisher Scientific, Waltham, MA, USA) was used for PCR product purification.

Host DNA was isolated from blood samples with a CWE9600 automatic nucleic acid extractor using a CWE9600 blood DNA kit following the manufacturer’s instructions and was genotyped on an Illumina Infinium Asian screening array (ASA-750K) following the Infinium HTS array protocol. The chip was developed by Novogene Bioinformatics Technology Co., Ltd., containing 738,980 sites in total, including the Illumina Asian screening array with an additional 50,000 SNP sites. All the samples with a call rate of >95% were reserved for further genotyping.

### Vaginal bacteriome data processing.

The original demultiplexed single-end 16S rRNA gene sequence data were preprocessed with the QIIME 2 Core 2020.8 distribution (53). All the reads were truncated to 370 nucleotides based on the quality scores that dropped at this number determined by reviewing the Interactive Quality Plot. All the truncated reads were then denoised and clustered by the DADA2 algorithm for quality control (QC) (54). Pretrained compatible classifiers based on the SILVA database (version 138) were downloaded from the official QIIME 2 website (https://docs.qiime2.org/2020.8/data-resources/) for the taxonomic assignments of denoised amplicon sequence variants (ASVs) (55, 56). To improve the taxonomic profiling, sequences that were resolved at the genus level but lacked a good resolution at the species level were queried through the BLAST database ref_prok_rep_genomes with local BLAST+ command-line applications (57, 58). BLAST best matches were considered hits with E values of <1 × 10^−50^, percentage of identical matches of >95%, and highest bit-score among each queried list. The lineages of queried taxa were extracted with the program from the “dib-lab/2018-ncbi-lineages” project on GitHub (https://github.com/dib-lab/2018-ncbi-lineages). Multiple or incompatible matches were left blank, except for *Lactobacillus paragasseri/gasseri* given its biological meaning in the classification of the vaginal bacterial community mentioned in previous publications (59, 60). The feature table was rarefied to the lowest number of read counts among the selected vaginal samples (3,128 reads). Taxa that were not assigned biologically meaningful names at any taxonomic level, had overall proportions below 0.01%, or were not bacteria were removed from the analysis. The databases used for the taxonomic analyses were deposited in Zenodo (http://doi.org/10.5281/zenodo.4480400). The MetaCyc pathway abundance was extrapolated from the original feature table by PICRUSt2 and then rarefied to the lowest level among the study samples (118,013, returns the largest integers not greater than 118,013.5; Fig. S2 at https://doi.org/10.5281/zenodo.5054434).

### Host genome data processing and QC.

All the genetic variants returned from the ASA chip were considered for the association analysis. Multiple duplicate loci with the same annotations were merged in SAS 9.4 (SAS Institute Inc.) prioritized by the locus with the highest Illumina GenTrain score. A comprehensive QC on SNPs and insertions/deletions (indels) was then performed with PLINK 1.9 (Fig. S3 at https://doi.org/10.5281/zenodo.5054434). Specifically, markers that met the following conditions were removed from the study: (i) missing genotypes for all samples, (ii) missingness rate across the individuals of >0.02, (iii) Hardy-Weinberg equilibrium (HWE) of *P* < 1 × 10^−5^, or (iv) minor allele frequency (MAF) of <0.01. The original chromosomal locations are based on chromosome build GRCh37/hg19. Sporadically missing genotypes of the SNPs across the host genome were imputed with BEAGLE 5.1 (61).

The copy number variations (CNVs) were detected by PennCNV 1.0.5 with the exclusion of loci with unrecognized genotypes (62). Sample QCs were performed with preset criteria including (i) the absolute value of waviness factor of >0.035, (ii) the standard deviation of logR ratio of >0.35, (iii) the number of CNV calls for a sample of >200, and (iv) the drift of B allele frequency of >0.01. The CNV QC process was performed for the remaining 311 participants who passed the sample QC with the filtration criteria including (i) the length of CNV calls of <50 bp and (ii) the fraction of gaps for merging two neighboring CNV calls of <0.2.

The QC process for host genetic variants generated 491,912 SNPs, 669 indels, and 6,859 CNVs across host genomes (Fig. S3 at https://doi.org/10.5281/zenodo.5054434). The density plots showed that most cleaned genotyped SNPs and indels were uniformly distributed on multiple chromosomes except for chromosome 6, where dense SNP markers were located in a region between the locations of 28 Mb and 56 Mb (Fig. S6 at https://doi.org/10.5281/zenodo.5054434).

### Bacteriome trait preparation and statistical analyses.

The overview of the vaginal bacteriome in pregnant women was described by the composition of vaginal community state types (CSTs) clustered by the partitioning around medoids (pam) algorithm based on a Bray-Curtis distance matrix. Pairwise taxonomic correlations at the genus and species levels were estimated by the SparCC algorithm using FastSpar 0.0.10 (63). The correlation coefficients were estimated as the average of 50 inference iterations refined by 10 exclusion iterations with the strength threshold of 0.1, and a sum of 2,000 bootstrap data sets was generated to calculate the empirical *P* values. Demographic covariates were compared among the CST groups, and different statistical tests were used with respect to their statistical types (Kruskal-Wallis test for continuous variables and Fisher’s exact tests for categorical variables). Pairwise comparisons were performed for those variables exhibiting a statistically significant global difference (*P* < 0.05). The two-sided *P* values were calculated for all the statistical tests.

The VBTs for the association analyses included the alpha diversity metrics, the beta diversity metrics, the binary and continuous traits (relative abundance [RAB]) of vaginal bacterial taxa, and the MetaCyc pathway abundance. All the VBTs were first processed with all the samples with available 16S amplicon data (*n* = 359) in order to make the most of the available information. Then, a sample with both host genome and 16S amplicon data (*n* = 315) was subsetted for the subsequent association analyses. For continuous (the alpha diversity metrics, the RAB of vaginal bacterial taxa, and the MetaCyc pathways) and multivariate (beta diversity) traits, the rank transformation (RNT) to normality was performed for the subsetted data with the rntransform() function within the R GenABEL package before fitting a linear model or a multivariate analysis of variance (MANOVA) (64).

All the alpha/beta diversity metrics were estimated by QIIME 2 using rarefaction data for the original ASV counts. The default alpha diversity metrics generated from the QIIME 2 core-metrics-phylogenetic pipeline were analyzed, including (i) ASV richness, as defined by the number of nonzero ASV counts observed; (ii) the Shannon index, a metric that measures both the number of ASVs and the inequality between ASV abundances; (iii) Faith phylogenetic diversity (Faith PD), a measure that uses phylogenetic distance to calculate diversity; and (iv) Pielou’s evenness, an index that measures diversity along with species richness. Beta diversity metrics including (i) Bray-Curtis dissimilarity, (ii) Unweighted UniFrac, and (iii) Weighted UniFrac were exported by the QIIME 2 diversity-lib plugin, and a 2-axis nonmetric multidimensional scaling (NMDS) analysis was performed for the exported beta diversity metrics with the metaMDS() function from the vegan package. The stresses of each NMDS beta diversity were 0.181 (Bray-Curtis dissimilarity), 0.162 (Unweighted UniFrac), and 0.086 (Weighted UniFrac), respectively.

For genetic association analysis with individual taxa, taxa were retained if they met the following criteria: (i) ≥5% of reads for at least one individual and (ii) ≥15% of participants with nonzero data (19). Additionally, the higher-level taxa that had an extremely high correlation (Spearman *r* > 0.985) with corresponding lower-level taxa were excluded from the association analyses to reduce the redundancy of phenotypic information (Fig. S4 at https://doi.org/10.5281/zenodo.5054434). This process generated 40 taxa for the subsequent association analyses with host genetic variants. Considering the unique nature of vaginal 16S rRNA gene data, both continuous and binary bacterial traits were of interest in this study (Fig. S5 at https://doi.org/10.5281/zenodo.5054434). The binary bacterial traits were considered either the presence or dominance of a retained taxon given its distribution across the study samples for 16S amplicon sequencing. Specifically, a taxon with zero counts in more than 5% of study samples was transformed to the presence/absence (P/A) trait and the analytic estimate reflects the association between host genetic variants and their presence; the dominance/nondominance (D/ND) of a given taxon was of interest for those taxa that had a dominant level of RAB (>90%) in more than 5% of study samples. Detailed information on phenotypic taxa and their phylogenetic structure is provided in the supplemental materials (Fig. S5 at https://doi.org/10.5281/zenodo.5054434).

For MetaCyc pathways, we retained those pathways that had (i) ≥5% of inferred abundances for at least one individual and (ii) ≥15% of participants with nonzero data. A total of 291 MetaCyc pathways were obtained for the association analysis with microbial function.

Primary association analyses for genetic variants with respect to multiple prepared VBTs were performed using either PLINK 1.9 (for SNPs and indels) or R software with linear regressions fitted for continuous traits (including all the alpha diversity metrics, the RAB of bacterial taxa, and the inferred MetaCyc pathways) and logistic regression fitted for binary traits (including P/A or D/ND of taxa given their distribution in study samples). The associations between host genetic variants and beta diversity metrics were performed using the manova() from the built-in R stat packages with dosage data processed by QCtools 2.0.8. An additive genetic model was assumed for the SNPs. Two types of covariates were considered for adjustment in the regression models: (i) demographic variables showing heterogeneity among the CST groups (global and pairwise comparison), first-time pregnancy status and baseline GA; (ii) variant-specific 10 genomic PCs for controlling the population structure in ancestry. Missing covariates were randomly imputed with the R mice package based on the observed data. Trait-specific associated loci with *P* of <1 × 10^−6^ are highlighted in the summary plots, and all the loci that reached a suggestive significance level (*P* < 1 × 10^−5^) are annotated with the closest genes using ANNOVAR in the supplemental tables (65). Further regional associations were assessed for those SNPs that reached the genome-wide significance level (*P* < 5 × 10^−8^) and had the lowest *P* values within a ±250-kb window by locuszoom and imputed 1000 Genomes Project (Phase 3) reference panel data from BEAGLE 5.1 (http://bochet.gcc.biostat.washington.edu/beagle/1000_Genomes_phase3_v5a/). Only the results with genomic inflation factor (GIF) λ of <1.1 were considered a subtle population structure and shown in the study (66, 67). The estimates for λ were either extracted from the PLINK log files or calculated by the estlambda() function from the R GenABEL package with the *method = ‘median’* option.

The narrow-sense genetic heritability using either SNPs or indels was estimated with covariates corrected for each VBT (including alpha diversity metrics, RAB, P/A and D/ND of bacterial taxa, and MetaCyc pathway abundances) by GCTA 1.93.1 using a genome-based restricted maximum-likelihood (GREML) method with a relatedness cutoff of 0.25 (68). The fixation index for SNPs was also estimated by the snpgdsFst() function from the R SNPRelate package for assessing the population differentiation with respect to each binary bacterial trait (69). Genetic structural correlations with VBTs were conducted using the extracted 10 principal components (PCs) for different types of genetic variants and were analyzed with a linear or logistic regression model or multivariate analysis of variance in line with the type of VBTs. The host genetic PCs were extracted by PLINK 1.9 with pruned genetic variants (SNPs and indels) in linkage disequilibrium (LD) as estimated by *r*^2^ > 0.8 in a 10-kb window, whereas the CNV-PCs were extracted by the psych package in the R program. Finally, joint association analyses for multiple VBTs were also performed with score tests from multivariate linear mixed models (mvLMM) using GEMMA (release 0.98.4) for all the genetic variants where the centered genotype relatedness matrix was estimated. Considering the computational efficiency and the biological implications of VBTs, two sets of multiple VBTs were considered for the joint association analyses: (i) a combination of the RAB of the top three bacterial taxa including Lactobacillus crispatus, Lactobacillus iners, and Gardnerella vaginalis and (ii) all the RAB of species-level taxa (16 taxa). The overall analytic approaches used for the association analyses were summarized in Table S1 at https://doi.org/10.5281/zenodo.5054434.

### Functional mapping and enrichment analyses.

Gene prioritization was based on a combination strategy of positional mapping and expression quantitative trait locus (eQTL) mapping in this study (70). For trait-specific associated genetic variants including SNPs and indels with a suggestive significant association (*P* < 1 × 10^−5^), the GTEx portal (version 8, dbGaP accession phs000424.v8.p2) was used to determine whether these associated variants were also eQTLs (71). Except for the prostate and testis, all the available tissues and SNP-gene-tissue pair results with a nominal *P* value less than 0.05 were considered eligible sites for eQTL mapping. Positionally associated genes with respect to each VBT were identified by MAGMA 1.09 (72). The SNPs or indels were annotated by positional mapping and with a generous gene definition that included 35 kb upstream and 10 kb downstream of each gene (73–75). The gene table was downloaded from the NCBI Entrez Gene database on 18 June 2020. Both the updated gene list and the GTEx data use the GRCh38/hg38 assembly with an incompatible coordinate for gene mapping. Therefore, the original coordinates were converted to the corresponding coordinates in GRCh38/hg38 using the UCSC liftOver (76). Summary statistics of SNPs/indels, irrespective of their significance level, were integrated into the MAGMA gene-based association analysis where both the mean and top models were implemented. In summary, the mapped genes for the subsequent enrichment analyses were considered those that (i) had an (eGene) *q* of <0.05 in GTEx eQTL mapping, (ii) had a raw *P* of <1 × 10^−5^ and *q* of <0.05 in MAGMA gene-based association analyses, (iii) were the nearest gene to a significant CNV (*P* < 1 × 10^−5^ and *q* < 0.05) within a ±5-Mb sliding window in the association analyses for individual VBTs, or (iv) were the closest gene of the SNPs/indels that reached the genome-wide significance level within a ±250-kb sliding window. Only the results with *λ* for genomic inflation of <1.1 were considered for the enrichment analyses.

All the mapped genes with available Entrez gene IDs were submitted to the GENE2FUNC pipeline with all the background genes and default parameters used in the FUMA platform (http://fuma.ctglab.nl/) to obtain an overview of the functionality of mapped genes in the biological context (70). The enrichment analysis for individual VBTs was locally analyzed by geneSCF v1.1 with the utilization of the GO_MF (molecular function), NCG, KEGG, and REACTOME databases (77). Differentially expressed genes (DEGs) against different databases were searched to find whether the obtained genes in relation to specific VBTs have any enrichment in molecular functions, different cancer types, or KEGG or REACTOME biological pathways.

### Data availability.

As this is not an international collaboration project, the data from human genetic resources are not publicly available due to the restrictions from the Regulation of the People’s Republic of China on the Administration of Human Genetic Resources. Other summary data in support of the findings of the study will be available upon request from the indicated corresponding author in compliance with the local laws and regulations (Yingjie Zheng, yjzheng@fudan.edu.cn). The databases used for the taxonomic analyses were deposited in Zenodo (http://doi.org/10.5281/zenodo.4480400).

## References

[1] Zheng DP, Liwinski T, Elinav E. 2020. Interaction between microbiota and immunity in health and disease. Cell Res 30:492–506. doi:10.1038/s41422-020-0332-7. 32433595 PMC7264227

[2] Lloyd-Price J, Mahurkar A, Rahnavard G, Crabtree J, Orvis J, Hall AB, Brady A, Creasy HH, McCracken C, Giglio MG, McDonald D, Franzosa EA, Knight R, White O, Huttenhower C. 2017. Strains, functions and dynamics in the expanded Human Microbiome Project. Nature 550:61–66. doi:10.1038/nature23889. 28953883 PMC5831082

[3] Wang J, Kurilshikov A, Radjabzadeh D, Turpin W, Croitoru K, Bonder MJ, Jackson MA, Medina-Gomez C, Frost F, Homuth G, Ruhlemann M, Hughes D, Kim HN, MiBioGen Consortium Initiative, Spector TD, Bell JT, Steves CJ, Timpson N, Franke A, Wijmenga C, Meyer K, Kacprowski T, Franke L, Paterson AD, Raes J, Kraaij R, Zhernakova A. 2018. Meta-analysis of human genome-microbiome association studies: the MiBioGen consortium initiative. Microbiome 6:101. doi:10.1186/s40168-018-0479-3. 29880062 PMC5992867

[4] Human Microbiome Project Consortium. 2012. A framework for human microbiome research. Nature 486:215–221. doi:10.1038/nature11209. 22699610 PMC3377744

[5] Chapman DK, Bartlett J, Powell J, Carter N. 2016. Bacterial vaginosis screening and treatment in pregnant women. J Midwifery Womens Health 61:628–631. doi:10.1111/jmwh.12475. 27383417

[6] Fettweis JM, Serrano MG, Brooks JP, Edwards DJ, Girerd PH, Parikh HI, Huang B, Arodz TJ, Edupuganti L, Glascock AL, Xu J, Jimenez NR, Vivadelli SC, Fong SS, Sheth NU, Jean S, Lee V, Bokhari YA, Lara AM, Mistry SD, Duckworth RA, Bradley SP, Koparde VN, Orenda XV, Milton SH, Rozycki SK, Matveyev AV, Wright ML, Huzurbazar SV, Jackson EM, Smirnova E, Korlach J, Tsai YC, Dickinson MR, Brooks JL, Drake JI, Chaffin DO, Sexton AL, Gravett MG, Rubens CE, Wijesooriya NR, Hendricks-Munoz KD, Jefferson KK, Strauss JF, Buck GA. 2019. The vaginal microbiome and preterm birth. Nat Med 25:1012–1021. doi:10.1038/s41591-019-0450-2. 31142849 PMC6750801

[7] Witkin SS, Mendes-Soares H, Linhares IM, Jayaram A, Ledger WJ, Forney LJ. 2013. Influence of vaginal bacteria and D- and L-lactic acid isomers on vaginal extracellular matrix metalloproteinase inducer: implications for protection against upper genital tract infections. mBio 4:e00460-13. doi:10.1128/mBio.00460-13. 23919998 PMC3735189

[8] Di Simone N, Santamaria Ortiz A, Specchia M, Tersigni C, Villa P, Gasbarrini A, Scambia G, D’Ippolito S. 2020. Recent insights on the maternal microbiota: impact on pregnancy outcomes. Front Immunol 11:528202. doi:10.3389/fimmu.2020.528202. 33193302 PMC7645041

[9] Mor G, Aldo P, Alvero AB. 2017. The unique immunological and microbial aspects of pregnancy. Nat Rev Immunol 17:469–482. doi:10.1038/nri.2017.64. 28627518

[10] Lewis FM, Bernstein KT, Aral SO. 2017. Vaginal microbiome and its relationship to behavior, sexual health, and sexually transmitted diseases. Obstet Gynecol 129:643–654. doi:10.1097/AOG.0000000000001932. 28277350 PMC6743080

[11] Gajer P, Brotman RM, Bai G, Sakamoto J, Schutte UM, Zhong X, Koenig SS, Fu L, Ma ZS, Zhou X, Abdo Z, Forney LJ, Ravel J. 2012. Temporal dynamics of the human vaginal microbiota. Sci Transl Med 4:132ra52. doi:10.1126/scitranslmed.3003605. PMC3722878 22553250

[12] Berman HL, McLaren MR, Callahan BJ. 2020. Understanding and interpreting community sequencing measurements of the vaginal microbiome. BJOG 127:139–146. doi:10.1111/1471-0528.15978. 31597208 PMC10801814

[13] Goodrich JK, Davenport ER, Clark AG, Ley RE. 2017. The relationship between the human genome and microbiome comes into view. Annu Rev Genet 51:413–433. doi:10.1146/annurev-genet-110711-155532. 28934590 PMC5744868

[14] Awany D, Allali I, Dalvie S, Hemmings S, Mwaikono KS, Thomford NE, Gomez A, Mulder N, Chimusa ER. 2018. Host and microbiome genome-wide association studies: current state and challenges. Front Genet 9:637. doi:10.3389/fgene.2018.00637. 30723493 PMC6349833

[15] Romero R, Hassan SS, Gajer P, Tarca AL, Fadrosh DW, Nikita L, Galuppi M, Lamont RF, Chaemsaithong P, Miranda J, Chaiworapongsa T, Ravel J. 2014. The composition and stability of the vaginal microbiota of normal pregnant women is different from that of non-pregnant women. Microbiome 2:4. doi:10.1186/2049-2618-2-4. 24484853 PMC3916806

[16] Suzuki TA, Ley RE. 2020. The role of the microbiota in human genetic adaptation. Science 370:eaaz6827. doi:10.1126/science.aaz6827. 33273073

[17] Blekhman R, Goodrich JK, Huang K, Sun Q, Bukowski R, Bell JT, Spector TD, Keinan A, Ley RE, Gevers D, Clark AG. 2015. Host genetic variation impacts microbiome composition across human body sites. Genome Biol 16:191. doi:10.1186/s13059-015-0759-1. 26374288 PMC4570153

[18] Bonder MJ, Kurilshikov A, Tigchelaar EF, Mujagic Z, Imhann F, Vila AV, Deelen P, Vatanen T, Schirmer M, Smeekens SP, Zhernakova DV, Jankipersadsing SA, Jaeger M, Oosting M, Cenit MC, Masclee AA, Swertz MA, Li Y, Kumar V, Joosten L, Harmsen H, Weersma RK, Franke L, Hofker MH, Xavier RJ, Jonkers D, Netea MG, Wijmenga C, Fu J, Zhernakova A. 2016. The effect of host genetics on the gut microbiome. Nat Genet 48:1407–1412. doi:10.1038/ng.3663. 27694959

[19] Hughes DA, Bacigalupe R, Wang J, Ruhlemann MC, Tito RY, Falony G, Joossens M, Vieira-Silva S, Henckaerts L, Rymenans L, Verspecht C, Ring S, Franke A, Wade KH, Timpson NJ, Raes J. 2020. Genome-wide associations of human gut microbiome variation and implications for causal inference analyses. Nat Microbiol 5:1079–1087. doi:10.1038/s41564-020-0743-8. 32572223 PMC7610462

[20] Kolde R, Franzosa EA, Rahnavard G, Hall AB, Vlamakis H, Stevens C, Daly MJ, Xavier RJ, Huttenhower C. 2018. Host genetic variation and its microbiome interactions within the Human Microbiome Project. Genome Med 10:6. doi:10.1186/s13073-018-0515-8. 29378630 PMC5789541

[21] Mehta SD, Nannini DR, Otieno F, Green SJ, Agingu W, Landay A, Zheng Y, Hou L. 2020. Host genetic factors associated with vaginal microbiome composition in Kenyan women. mSystems 5:e00502-20. doi:10.1128/mSystems.00502-20. 32723796 PMC7394359

[22] Genc MR, Vardhana S, Delaney ML, Onderdonk A, Tuomala R, Norwitz E, Witkin SS, The MAP Study Group. 2004. Relationship between a toll-like receptor-4 gene polymorphism, bacterial vaginosis-related flora and vaginal cytokine responses in pregnant women. Eur J Obstet Gynecol Reprod Biol 116:152–156. doi:10.1016/j.ejogrb.2004.02.010. 15358455

[23] Jones NM, Holzman C, Friderici KH, Jernigan K, Chung H, Wirth J, Fisher R. 2010. Interplay of cytokine polymorphisms and bacterial vaginosis in the etiology of preterm delivery. J Reprod Immunol 87:82–89. doi:10.1016/j.jri.2010.06.158. 20965572 PMC3005194

[24] Doerflinger SY, Throop AL, Herbst-Kralovetz MM. 2014. Bacteria in the vaginal microbiome alter the innate immune response and barrier properties of the human vaginal epithelia in a species-specific manner. J Infect Dis 209:1989–1999. doi:10.1093/infdis/jiu004. 24403560

[25] Rocha J, Botelho J, Ksiezarek M, Perovic SU, Machado M, Carrico JA, Pimentel LL, Salsinha S, Rodriguez-Alcala LM, Pintado M, Ribeiro TG, Peixe L. 2020. Lactobacillus mulieris sp. nov., a new species of Lactobacillus delbrueckii group. Int J Syst Evol Microbiol 70:1522–1527. doi:10.1099/ijsem.0.003901. 31951193

[26] Putonti C, Shapiro JW, Ene A, Tsibere O, Wolfe AJ. 2020. Comparative genomic study of Lactobacillus jensenii and the newly defined Lactobacillus mulieris species identifies species-specific functionality. mSphere 5:e00560-20. doi:10.1128/mSphere.00560-20. 32817455 PMC7426171

[27] Demmitt BA, Corley RP, Huibregtse BM, Keller MC, Hewitt JK, McQueen MB, Knight R, McDermott I, Krauter KS. 2017. Genetic influences on the human oral microbiome. BMC Genomics 18:659. doi:10.1186/s12864-017-4008-8. 28836939 PMC5571580

[28] Smith SB, Ravel J. 2017. The vaginal microbiota, host defence and reproductive physiology. J Physiol 595:451–463. doi:10.1113/JP271694. 27373840 PMC5233653

[29] Larsen B, Monif GR. 2001. Understanding the bacterial flora of the female genital tract. Clin Infect Dis 32:e69–e77. doi:10.1086/318710. 11181139

[30] Srinivasan S, Fredricks DN. 2008. The human vaginal bacterial biota and bacterial vaginosis. Interdiscip Perspect Infect Dis 2008:750479. doi:10.1155/2008/750479. 19282975 PMC2648628

[31] Patterson JL, Stull-Lane A, Girerd PH, Jefferson KK. 2010. Analysis of adherence, biofilm formation and cytotoxicity suggests a greater virulence potential of Gardnerella vaginalis relative to other bacterial-vaginosis-associated anaerobes. Microbiology (Reading) 156:392–399. doi:10.1099/mic.0.034280-0. 19910411 PMC2890091

[32] Zhou X, Michal JJ, Zhang L, Ding B, Lunney JK, Liu B, Jiang Z. 2013. Interferon induced IFIT family genes in host antiviral defense. Int J Biol Sci 9:200–208. doi:10.7150/ijbs.5613. 23459883 PMC3584916

[33] Pidugu VK, Pidugu HB, Wu MM, Liu CJ, Lee TC. 2019. Emerging functions of human IFIT proteins in cancer. Front Mol Biosci 6:148. doi:10.3389/fmolb.2019.00148. 31921891 PMC6930875

[34] Fensterl V, Sen GC. 2015. Interferon-induced Ifit proteins: their role in viral pathogenesis. J Virol 89:2462–2468. doi:10.1128/JVI.02744-14. 25428874 PMC4325746

[35] Der SD, Zhou A, Williams BR, Silverman RH. 1998. Identification of genes differentially regulated by interferon alpha, beta, or gamma using oligonucleotide arrays. Proc Natl Acad Sci U S A 95:15623–15628. doi:10.1073/pnas.95.26.15623. 9861020 PMC28094

[36] Kohli A, Zhang X, Yang J, Russell RS, Donnelly RP, Sheikh F, Sherman A, Young H, Imamichi T, Lempicki RA, Masur H, Kottilil S. 2012. Distinct and overlapping genomic profiles and antiviral effects of Interferon-lambda and -alpha on HCV-infected and noninfected hepatoma cells. J Viral Hepat 19:843–853. doi:10.1111/j.1365-2893.2012.01610.x. 23121362 PMC3511888

[37] Tang J, Wu X, Mou M, Wang C, Wang L, Li F, Guo M, Yin J, Xie W, Wang X, Wang Y, Ding Y, Xue W, Zhu F. 2021. GIMICA: host genetic and immune factors shaping human microbiota. Nucleic Acids Res 49:D715–D722. doi:10.1093/nar/gkaa851. 33045729 PMC7779047

[38] Gutierrez-Merino J, Isla B, Combes T, Martinez-Estrada F, Maluquer De Motes C. 2020. Beneficial bacteria activate type-I interferon production via the intracellular cytosolic sensors STING and MAVS. Gut Microbes 11:771–788. doi:10.1080/19490976.2019.1707015. 31941397 PMC7524384

[39] John SP, Sun J, Carlson RJ, Cao B, Bradfield CJ, Song J, Smelkinson M, Fraser IDC. 2018. IFIT1 exerts opposing regulatory effects on the inflammatory and interferon gene programs in LPS-activated human macrophages. Cell Rep 25:95–106.e6. doi:10.1016/j.celrep.2018.09.002. 30282041 PMC6492923

[40] Barton PT, Gerber S, Skupski DW, Witkin SS. 2003. Interleukin-1 receptor antagonist gene polymorphism, vaginal interleukin-1 receptor antagonist concentrations, and vaginal ureaplasma urealyticum colonization in pregnant women. Infect Immun 71:271–274. doi:10.1128/IAI.71.1.271-274.2003. 12496176 PMC143390

[41] Genc MR, Onderdonk AB, Vardhana S, Delaney ML, Norwitz ER, Tuomala RE, Paraskevas LR, Witkin SS, the MAP Study Group. 2004. Polymorphism in intron 2 of the interleukin-1 receptor antagonist gene, local midtrimester cytokine response to vaginal flora, and subsequent preterm birth. Am J Obstet Gynecol 191:1324–1330. doi:10.1016/j.ajog.2004.05.074. 15507961

[42] Goepfert AR, Varner M, Ward K, Macpherson C, Klebanoff M, Goldenberg RL, Mercer B, Meis P, Iams J, Moawad A, Carey JC, Leveno K, Wapner R, Caritis SN, Miodovnik M, Sorokin Y, O’Sullivan MJ, Van Dorsten JP, Langer O, NICHD Maternal-Fetal Medicine Units Network. 2005. Differences in inflammatory cytokine and Toll-like receptor genes and bacterial vaginosis in pregnancy. Am J Obstet Gynecol 193:1478–1485. doi:10.1016/j.ajog.2005.03.053. 16202743

[43] Si J, You HJ, Yu J, Sung J, Ko G. 2017. Prevotella as a hub for vaginal microbiota under the influence of host genetics and their association with obesity. Cell Host Microbe 21:97–105. doi:10.1016/j.chom.2016.11.010. 28017660

[44] Ivashkiv LB, Donlin LT. 2014. Regulation of type I interferon responses. Nat Rev Immunol 14:36–49. doi:10.1038/nri3581. 24362405 PMC4084561

[45] Abrahams VM, Mor G. 2005. Toll-like receptors and their role in the trophoblast. Placenta 26:540–547. doi:10.1016/j.placenta.2004.08.010. 15993703

[46] Yano J, Lilly E, Barousse M, Fidel PL, Jr. 2010. Epithelial cell-derived S100 calcium-binding proteins as key mediators in the hallmark acute neutrophil response during Candida vaginitis. Infect Immun 78:5126–5137. doi:10.1128/IAI.00388-10. 20823201 PMC2981313

[47] Falconer DS, Mackay TFC. 1996. Introduction to quantitative genetics, 4th ed. Pearson Prentice Hall, Harlow, England.

[48] Davenport ER. 2016. Elucidating the role of the host genome in shaping microbiome composition. Gut Microbes 7:178–184. doi:10.1080/19490976.2016.1155022. 26939746 PMC4856462

[49] Fisher RA. 1958. The genetical theory of natural selection. Dover Publications, New York, NY.

[50] Hoffmann AA, Merila J, Kristensen TN. 2016. Heritability and evolvability of fitness and nonfitness traits: lessons from livestock. Evolution 70:1770–1779. doi:10.1111/evo.12992. 27346243

[51] Gustafsson L. 1986. Lifetime reproductive success and heritability: empirical support for Fisher’s fundamental theorem. Am Nat 128:761–764. doi:10.1086/284601.

[52] Mousseau TA, Roff DA. 1987. Natural selection and the heritability of fitness components. Heredity (Edinb) 59:181–197. doi:10.1038/hdy.1987.113. 3316130

[53] Bolyen E, Rideout JR, Dillon MR, Bokulich N, Abnet CC, Al-Ghalith GA, Alexander H, Alm EJ, Arumugam M, Asnicar F, Bai Y, Bisanz JE, Bittinger K, Brejnrod A, Brislawn CJ, Brown CT, Callahan BJ, Caraballo-Rodriguez AM, Chase J, Cope EK, Da Silva R, Diener C, Dorrestein PC, Douglas GM, Durall DM, Duvallet C, Edwardson CF, Ernst M, Estaki M, Fouquier J, Gauglitz JM, Gibbons SM, Gibson DL, Gonzalez A, Gorlick K, Guo JR, Hillmann B, Holmes S, Holste H, Huttenhower C, Huttley GA, Janssen S, Jarmusch AK, Jiang LJ, Kaehler BD, Bin Kang K, Keefe CR, Keim P, Kelley ST, Knights D, et al. 2019. Reproducible, interactive, scalable and extensible microbiome data science using QIIME 2. Nat Biotechnol 37:852–857. doi:10.1038/s41587-019-0209-9. 31341288 PMC7015180

[54] Callahan BJ, McMurdie PJ, Rosen MJ, Han AW, Johnson AJ, Holmes SP. 2016. DADA2: high-resolution sample inference from Illumina amplicon data. Nat Methods 13:581–583. doi:10.1038/nmeth.3869. 27214047 PMC4927377

[55] Bokulich NA, Robeson M, Dillon MR. 2020. bokulich-lab/RESCRIPt. Zenodo. doi:10.5281/zenodo.3891931.

[56] Bokulich NA, Kaehler BD, Rideout JR, Dillon M, Bolyen E, Knight R, Huttley GA, Gregory Caporaso J. 2018. Optimizing taxonomic classification of marker-gene amplicon sequences with QIIME 2’s q2-feature-classifier plugin. Microbiome 6:90. doi:10.1186/s40168-018-0470-z. 29773078 PMC5956843

[57] National Center for Biotechnology Information. 2008. BLAST command line applications user manual. National Center for Biotechnology Information, Bethesda, MD. https://www.ncbi.nlm.nih.gov/books/NBK279690/.

[58] Ren YJ, Su H, She YL, Dai CY, Xie D, Narrandes S, Huang SJ, Chen C, Xu WN. 2019. Whole genome sequencing revealed microbiome in lung adenocarcinomas presented as ground-glass nodules. Transl Lung Cancer Res 8:235–246. doi:10.21037/tlcr.2019.06.11. 31367537 PMC6626866

[59] Mehta O, Ghosh TS, Kothidar A, Gowtham MR, Mitra R, Kshetrapal P, Wadhwa N, Thiruvengadam R, GARBH-Ini study group, Nair GB, Bhatnagar S, Das B. 2020. Vaginal microbiome of pregnant Indian women: insights into the genome of dominant Lactobacillus species. Microb Ecol 80:487–499. doi:10.1007/s00248-020-01501-0. 32206831

[60] Ravel J, Gajer P, Abdo Z, Schneider GM, Koenig SSK, McCulle SL, Karlebach S, Gorle R, Russell J, Tacket CO, Brotman RM, Davis CC, Ault K, Peralta L, Forney LJ. 2011. Vaginal microbiome of reproductive-age women. Proc Natl Acad Sci U S A 108:4680–4687. doi:10.1073/pnas.1002611107. 20534435 PMC3063603

[61] Browning BL, Zhou Y, Browning SR. 2018. A one-penny imputed genome from next-generation reference panels. Am J Hum Genet 103:338–348. doi:10.1016/j.ajhg.2018.07.015. 30100085 PMC6128308

[62] Fang L, Wang K. 2018. Identification of copy number variants from SNP arrays using PennCNV. Methods Mol Biol 1833:1–28. doi:10.1007/978-1-4939-8666-8_1. 30039360

[63] Watts SC, Ritchie SC, Inouye M, Holt KE. 2019. FastSpar: rapid and scalable correlation estimation for compositional data. Bioinformatics 35:1064–1066. doi:10.1093/bioinformatics/bty734. 30169561 PMC6419895

[64] Aulchenko YS, Ripke S, Isaacs A, van Duijn CM. 2007. GenABEL: an R library for genome-wide association analysis. Bioinformatics 23:1294–1296. doi:10.1093/bioinformatics/btm108. 17384015

[65] Wang K, Li M, Hakonarson H. 2010. ANNOVAR: functional annotation of genetic variants from high-throughput sequencing data. Nucleic Acids Res 38:e164. doi:10.1093/nar/gkq603. 20601685 PMC2938201

[66] Piras IS, Perdigones N, Zismann V, Briones N, Facista S, Rivera JL, Rozanski E, London CA, Hendricks WPD. 2020. Identification of genetic susceptibility factors associated with canine gastric dilatation-volvulus. Genes (Basel) 11:1313. doi:10.3390/genes11111313. PMC7694454 33167491

[67] Yang J, Weedon MN, Purcell S, Lettre G, Estrada K, Willer CJ, Smith AV, Ingelsson E, O’Connell JR, Mangino M, Magi R, Madden PA, Heath AC, Nyholt DR, Martin NG, Montgomery GW, Frayling TM, Hirschhorn JN, McCarthy MI, Goddard ME, Visscher PM, GIANT Consortium. 2011. Genomic inflation factors under polygenic inheritance. Eur J Hum Genet 19:807–812. doi:10.1038/ejhg.2011.39. 21407268 PMC3137506

[68] Yang J, Lee SH, Goddard ME, Visscher PM. 2011. GCTA: a tool for genome-wide complex trait analysis. Am J Hum Genet 88:76–82. doi:10.1016/j.ajhg.2010.11.011. 21167468 PMC3014363

[69] Wright S. 1951. The genetical structure of populations. Ann Eugen 15:323–354. doi:10.1111/j.1469-1809.1949.tb02451.x. 24540312

[70] Watanabe K, Taskesen E, van Bochoven A, Posthuma D. 2017. Functional mapping and annotation of genetic associations with FUMA. Nat Commun 8:1826. doi:10.1038/s41467-017-01261-5. 29184056 PMC5705698

[71] GTEx Consortium. 2015. Human genomics. The Genotype-Tissue Expression (GTEx) pilot analysis: multitissue gene regulation in humans. Science 348:648–660. doi:10.1126/science.1262110. 25954001 PMC4547484

[72] de Leeuw CA, Mooij JM, Heskes T, Posthuma D. 2015. MAGMA: generalized gene-set analysis of GWAS data. PLoS Comput Biol 11:e1004219. doi:10.1371/journal.pcbi.1004219. 25885710 PMC4401657

[73] Pardiñas AF, Holmans P, Pocklington AJ, Escott-Price V, Ripke S, Carrera N, Legge SE, Bishop S, Cameron D, Hamshere ML, Han J, Hubbard L, Lynham A, Mantripragada K, Rees E, MacCabe JH, McCarroll SA, Baune BT, Breen G, Byrne EM, Dannlowski U, Eley TC, Hayward C, Martin NG, McIntosh AM, Plomin R, Porteous DJ, Wray NR, Caballero A, Geschwind DH, Huckins LM, Ruderfer DM, Santiago E, Sklar P, Stahl EA, Won H, Agerbo E, Als TD, Andreassen OA, Bækvad-Hansen M, Mortensen PB, Pedersen CB, Børglum AD, Bybjerg-Grauholm J, Djurovic S, Durmishi N, Pedersen MG, Golimbet V, Grove J, Hougaard DM, et al. 2018. Common schizophrenia alleles are enriched in mutation-intolerant genes and in regions under strong background selection. Nat Genet 50:381–389. doi:10.1038/s41588-018-0059-2. 29483656 PMC5918692

[74] Sey NYA, Hu B, Mah W, Fauni H, McAfee JC, Rajarajan P, Brennand KJ, Akbarian S, Won H. 2020. A computational tool (H-MAGMA) for improved prediction of brain-disorder risk genes by incorporating brain chromatin interaction profiles. Nat Neurosci 23:583–593. doi:10.1038/s41593-020-0603-0. 32152537 PMC7131892

[75] Network and Pathway Analysis Subgroup of Psychiatric Genomics Consortium. 2015. Psychiatric genome-wide association study analyses implicate neuronal, immune and histone pathways. Nat Neurosci 18:199–209. doi:10.1038/nn.3922. 25599223 PMC4378867

[76] Bioconductor Package Maintainer. 2020. liftOver: changing genomic coordinate systems with rtracklayer::liftOver. https://www.bioconductor.org/help/workflows/liftOver/.

[77] Subhash S, Kanduri C. 2016. GeneSCF: a real-time based functional enrichment tool with support for multiple organisms. BMC Bioinformatics 17:365. doi:10.1186/s12859-016-1250-z. 27618934 PMC5020511

